# Multi-omic insights of preeclampsia and cardiovascular health outcomes

**DOI:** 10.1038/s43856-025-01165-2

**Published:** 2025-10-30

**Authors:** Emma M. Giesen, Stefan Verlohren, Sandra M. Blois

**Affiliations:** 1 University-Medical Centre Hamburg-Eppendorf, Hamburg, Germany; 2 Glyco-HAM, a Cooperation of Universität Hamburg, Technology Platforms Spectrometry and University Medical Center Hamburg-Eppendorf, Hamburg, Germany

**Keywords:** Cardiovascular diseases, Reproductive disorders

## Abstract

Preeclampsia is a complex, hypertensive pregnancy disorder which is linked to an increased cardiovascular disease risk in both mothers and their offspring. Maternal haemodynamic adaptation during gestation is essential for adequate foetal nutrient supply and development, fundamentally shaping the offspring’s cardiovascular health trajectory. In preeclampsia, this process is disrupted, leading to lasting effects for both the mother and the child. While the clinical features of preeclampsia in this context have been subject to extensive investigation, it remains a critical challenge to elucidate the molecular mechanisms underpinning its long-term cardiovascular consequences. This review synthesises multi-omics, including genomics, epigenomics, transcriptomics and metabolomics, and systems-biology insights to elucidate mechanisms, identify candidate biomarkers, and shape personalised medicine approaches. By bridging molecular and clinical understanding, we discuss how these approaches uncover prenatal adversity from preeclampsia exposure influencing cardiovascular disease risk in mothers and offspring, offering a roadmap to improve long-term cardiovascular outcomes.

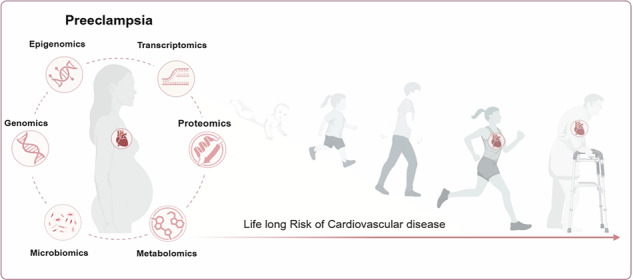

## Abstract (web summary)

Giesen et al. review multi-omics and systems-biology approaches to investigate how preeclampsia disrupts maternal haemodynamics, drives early life-adversity and influences cardiovascular risk in mothers and their offspring. They identify underlying molecular mechanisms and candidate biomarkers that may inform personalised medicine frameworks.

## Introduction

Preeclampsia (PE) is a multisystem pregnancy disorder, complicating 3–5% of all pregnancies^1^. It is one of the leading causes of maternal and perinatal morbidity, especially in low- and middle-income countries^1^. Manifesting after the 20th week of gestation, PE is characterised by the emergence of new-onset hypertension, defined as a systolic blood pressure of 140 mmHg or greater and a diastolic blood pressure above 90 mmHg, accompanied by proteinuria, which refers to high levels of protein in the urine, or other indicators of end-organ dysfunction. Consequently, careful monitoring and management are essential to mitigate risk to both maternal and foetal health^2,3^. Accordingly, PE is commonly classified by onset timing into early- (<34 weeks of gestation; EO-PE) and late- (>34 weeks of gestation; LO-PE) onset PE to estimate outcomes and associated risk factors. While this distinction captures key clinical features, it fails to reflect the underlying biological complexity^4^. In EO-PE, the placenta seems to play a critical role since disrupted remodelling of the spiral arteries causes placental hypoperfusion and hypoxia, which induces oxidative stress, triggering a heightened systemic inflammatory response. Subsequently, endothelial dysfunction and vasoconstriction develop, contributing to the onset of systemic hypertension and end-organ hypoperfusion^5^. As a consequence, there’s a release of a variety of anti-angiogenic factors, substances that inhibit the formation of new blood vessels, including soluble Fms-like tyrosine kinase-1 (sFlt-1), soluble endoglin and other cytokines and oxidants^6^. Despite its relevance, this model does not fully capture the full spectrum of PE, particularly in LO-PE cases where maternal factors are considered to be the primary drivers of the disease^7^. Apart from impaired placental function, various mechanisms such as angiogenic imbalance^8^, oxidative stress^6,9^, immunological causes^10^, maternal metabolic status^11^ and inadequate cardiovascular adaptation^12,13^, underlie its pathogenesis. Its heterogeneous nature additionally complicates both diagnosis and management^14,15^. PE can persist after the delivery of the placenta, including the development of de novo postpartum PE, further highlighting the inadequacy of a timing-based framework^15^.

PE’s impact extends beyond pregnancy, as placental delivery does not alleviate the heightened susceptibility to other illnesses^16^. One of the most significant short- and long-term health complications is an increased risk of cardiovascular disease (CVD) in the mother and is increasingly recognised as a contributor to cardiovascular changes in the offspring^17,18^. After a preeclamptic pregnancy, women have a 4-times higher risk of developing heart failure (HF) and a 2-times elevated risk of coronary heart disease, stroke, and death from cardiovascular issues^19^. Both CVD and PE share a plethora of risk factors, including obesity, insulin resistance, dyslipidaemia, heightened inflammatory responses, hypercoagulable state, endothelial dysfunction^19^ and gut dysbiosis^20–22^. Pregnancy can be regarded as a physiological stress test revealing a predisposition to diseases that might otherwise remain hidden for years. In this context, it remains unresolved whether PE represents an independent risk factor for CVD or reflects an early manifestation of pre-existing cardiovascular susceptibility. Maternal physiological and molecular adaptations during PE establish an altered intrauterine environment, which profoundly influences foetal programming ^23^. Although the extent of this early life adversity for the offspring is not fully understood, increasing evidence implicates PE in shaping prolonged cardiovascular trajectories in the offspring. Children of mothers with PE exhibit CVD risk factors throughout childhood and young adulthood^24^. Nonetheless, the mechanisms by which in utero exposure to PE disrupts cardiovascular development and the molecular pathways underlying long-term cardiovascular sequelae in both mother and offspring are not yet fully understood.

This review examines how advances in omics and systems biology may uncover the molecular mechanisms underpinning PE. By recognising the dynamic interplay between mother and foetus in PE pathogenesis, this review highlights integrated molecular signatures and bidirectional mechanisms shaping foetal development and CVD risk. A deeper understanding of these interconnected pathways of PE exposure may pave the way for a framework that integrates personalised risk profiles and health trajectories of the mother and offspring to improve adverse cardiovascular outcomes.

## Adaptations during pregnancy: shared pathophysiological mechanisms of PE and CVD

### Cardiac physiology in pregnancy and PE

**Fig. 1:**
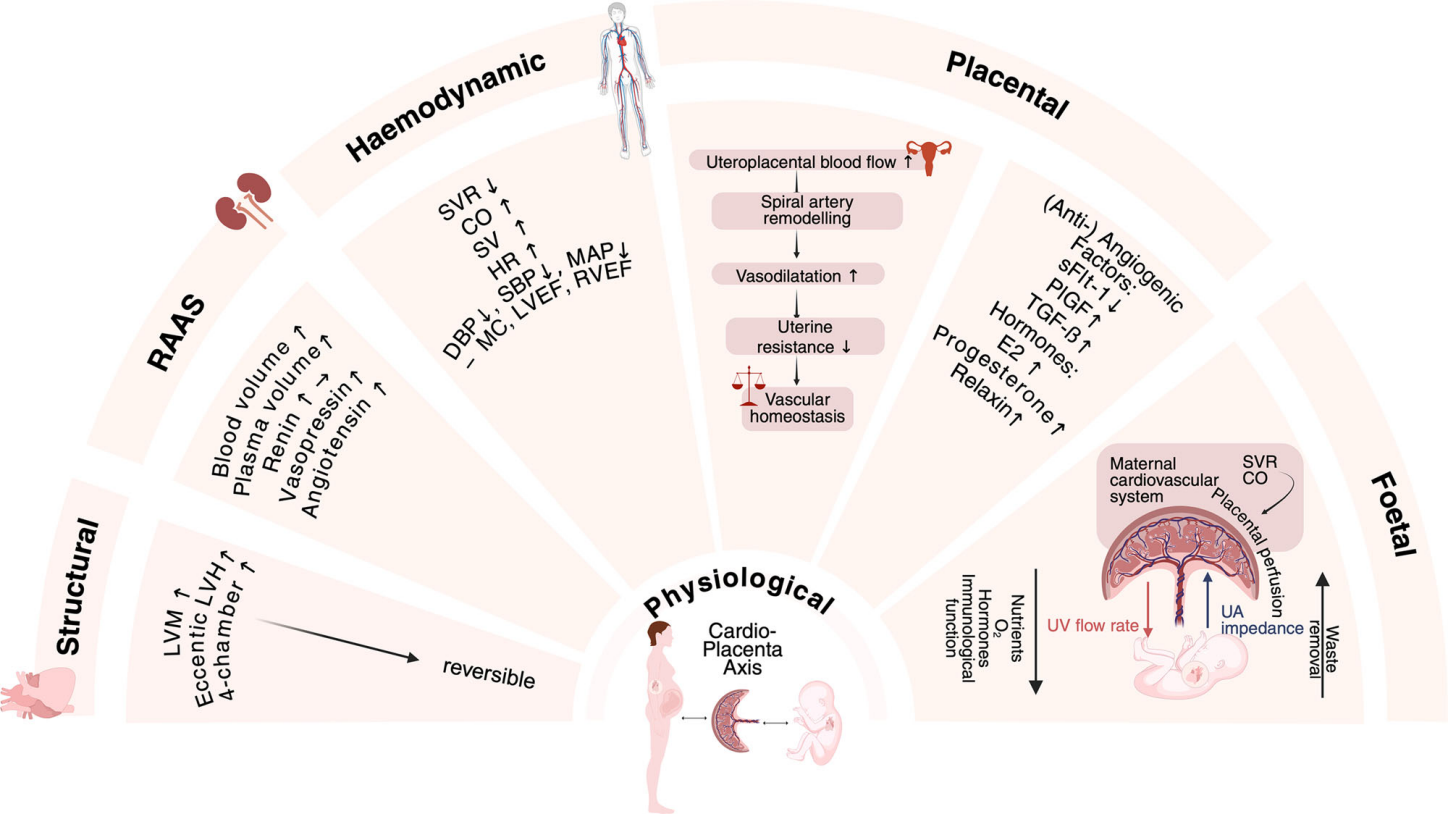
Physiological cardiovascular adaptation in pregnancy^46,47,53,74,161–184^. In a physiological pregnancy, maternal cardiac output increases, driven by increased blood volume and heart rate, while systemic vascular resistance decreases, enhancing uteroplacental blood flow. Spiral artery remodelling converts the uterine vasculature into wide, low-resistance channels, permitting sustained high-volume maternal blood flow to the placenta. This facilitates effective transfer of oxygen and nutrients, supporting foetal development and growth. CO Cardiac Output, DBP Diastolic Blood Pressure, E2 Oestrogen, HR Heart Rate, LVEF Left Ventricular Ejection Fraction, LVH Left Ventricular Hypertrophy, LVM Left Ventricular Mass, LVW Left Ventricular Wall, MC Myocardial Contractility, RAAS Renin-Angiotensin-Aldosterone-System, RVEF Right Ventricular Ejection Fraction, SBP Systolic Blood Pressure, sFlt-1 Soluble FMS-Like Tyrosine Kinase-1, SV Stroke Volume, SVR Systemic Vascular Resistance, TGF-ß Transforming Growth Factor-Beta, O2 Oxygen.

**Fig. 2:**
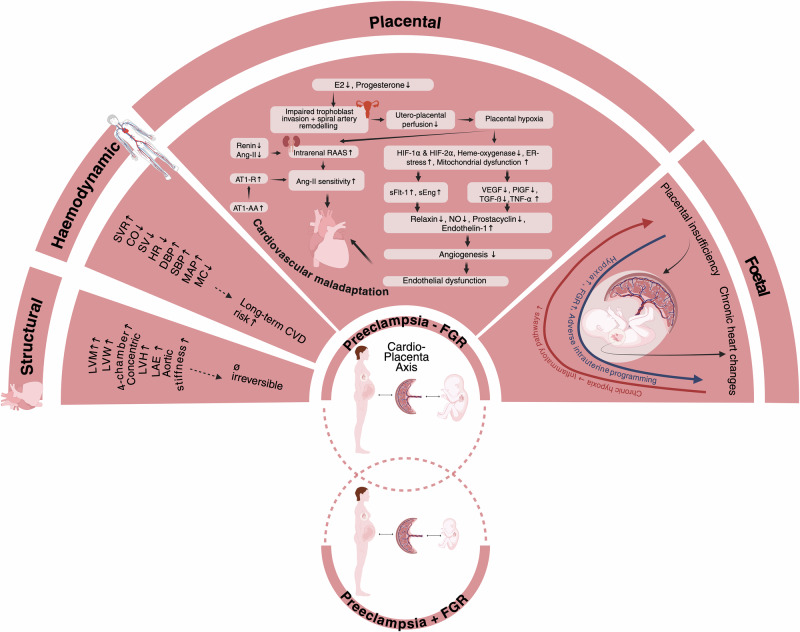
Cardiovascular maladaptation in preeclampsia^46,47,53,74,161–184^. In PE, the maternal cardiovascular system exhibits maladaptive changes, including increased cardiac afterload, diastolic dysfunction, and vascular remodelling, some of which may persist postpartum. These alterations are associated with an elevated lifetime risk of cardiovascular disease and may influence foetal cardiovascular programming. Endothelial dysfunction contributes to increased systemic vascular resistance and impaired uteroplacental perfusion. Concurrently, defective remodelling of the spiral arteries results in high-resistance placental circulation, reducing oxygen and nutrient transfer potentially compromising foetal growth trajectories. Ang-II Angiotensin-II, AT1-AA Angiotensin-II Type 1 Receptor-Autoantibodies, AT1-R Angiotensin II Type 1-Receptor, CO Cardiac Output, DBP Diastolic Blood Pressure, E2 Oestrogen, FGR Foetal Growth Restriction, HR Heart Rate, LAE Left Arterial Enlargement, LVEF Left Ventricular Ejection Fraction, LVH Left Ventricular Hypertrophy, LVM Left Ventricular Mass, LVW Left Ventricular Wall, MAP Mean Arterial Pressure, MC Myocardial Contractility, NO Nitric Oxide, RAAS Renin-Angiotensin-Aldosterone-System, RVEF Right Ventricular Ejection Fraction, SBP Systolic Blood Pressure, sFlt-1 Soluble FMS-Like Tyrosine Kinase-1, SV Stroke Volume, SVR Systemic Vascular Resistance, TGF-ß Transforming Growth Factor-Beta, TNF-α Tumour Necrosis Factor-Alpha, VEGF Vascular Endothelial Growth Factor, O2 Oxygen.

Pregnancy requires specific cardiovascular adaptations to ensure sufficient perfusion of the placenta (Fig. 1). Physiologically, the hormonal changes of pregnancy are accompanied by increased maternal blood volume and cardiac output, indicative of the necessity of structural adaptation^25^. This process can be a stress test for the cardiovascular system of the mother and can lead to the mother being susceptible to developing PE. With PE, alterations in global left ventricular function and geometry are often observed during the acute phase. These subclinical changes are recognised as important indicators for cardiovascular risk stratification^26^. In preeclamptic pregnancies, this physiological cardiovascular adaptation process is disrupted beyond pregnancy (Fig. 2). There is growing evidence supporting the persistence of pathological remodelling processes postpartum. In a study conducted by Garrido-Gimenez et al. women with a preeclamptic history had a higher prevalence of hypertensive disorders and dyslipidaemia than women without PE^27^. Furthermore, women with a preeclamptic history have a 2-fold higher prevalence of left ventricular hypertrophy compared with women from the general population, independently of cardiovascular risk factors^28^.

Cardiovascular risk stratification in PE is commonly aligned with EO-PE and LO-PE. In a clinical study, Hauge et al. corroborated that women with EO-PE are more likely to be predisposed to hypertension (51.1% vs. 35.1%, P $\le$ 0.001) and had a higher prevalence of atherosclerosis (28.8% vs 22.2%, respectively; *P* = 0.088; adjusted odds ratio, 1.74; 95% confidence interval, 1.01–3.01; *P* = 0.045 after adjustment for maternal age at index pregnancy, pre-pregnancy body mass index (BMI), parity, diabetes in pregnancy, smoking in pregnancy, offspring birthweight and sex, and follow-up length) compared to women with LO-PE^29^. EO-PE is considered to be of placental origin and has been linked to abnormal uterine Doppler, foetal growth restriction (FGR), and adverse maternal and neonatal outcomes^30^. In contrast, LO-PE, the most common form of PE, is believed to be mostly associated with maternal risk factors like maternal age $\ge$ 35 years, history of previous PE, cardiac disease, chronic hypertension, increased maternal BMI and other symptoms of metabolic syndrome^7,31^. This condition is associated with a normal or slightly increased uterine resistance index, low rates of foetal involvement and more favourable outcomes^30^. Among women with PE, those presenting with FGR demonstrate the most severe cardiovascular manifestations. This is characterised by reduced cardiac output and elevated systemic vascular resistance, irrespective of the timing of onset during gestation^32–34^. Therefore, the current classification of LO-PE and EO-PE may require further refinement. Additionally, in women with congenital heart defects, placental abnormalities such as growth disturbances, PE and preterm birth can be observed more frequently, suggesting a relationship between impaired placentation and cardiovascular health^35^. Women who subsequently develop PE/FGR exhibit lower preconception cardiac output (4.9 L/min vs. 5.8 L/min, *P* = 0.002) and cardiac index, while mean arterial pressure and total peripheral resistance are significantly higher^34^. Offspring birthweight at term positively correlated with maternal prepregnancy cardiac output^34^. While low birth weight has been linked to an increased risk of coronary heart disease in the offspring later in life^23^, it has also been associated with a higher risk of ischaemic heart disease in the mother^36^.

These findings underscore the intricate relationship between maternal cardiovascular health and placental function in ensuring healthy foetal growth and development^37,38^. Omics approaches can aid in further elucidating the molecular pathophysiology underlying these clinical observations of placental dysfunction. Defining molecular subtypes and their connection to CVD may improve risk stratification and facilitate early interventions for women with PE who are at elevated cardiovascular risk while supporting cardiovascular trajectories for their children from the earliest stages of life.

### Vascular pathophysiology in PE

During pregnancy, the peripheral vasculature, along with the heart, is subject to significant changes. Peripheral vasodilatation precedes full placentation and the development of the uteroplacental architecture, accompanied by renal vasodilatation and an activation of the renin-angiotensin-aldosterone system (RAAS)^39^. The RAAS plays an important role in the regulation of blood pressure and electrolyte homoeostasis and is a pharmacological target for antihypertensive medication. Under pathophysiological conditions, the RAAS is a key driver of inflammation and structural changes, processes with a critical role in the onset and progression of CVDs like atherosclerosis, hypertension, myocardial infarction and HF^40^. During pregnancy, several key RAAS components regulate essential physiological processes in both the mother and the foetus. Angiotensinogen, the upstream substrate of the renin-angiotensin cascade, is a key determinant of the angiotensin peptide generation and has been linked to PE^41^. A study identified angiotensin as a central regulator of placental transcriptional networks in PE, with extensive connections to differentially expressed genes, including *FLT1* and *LEP*, which encode s-Flt1 and leptin, respectively, and showed enrichment in the renin-angiotensin signalling pathway^14^. These proteins contribute to placental dysfunction: sFlt-1 sequesters vascular endothelial growth factor and placental growth factor to impair placental angiogenesis, while leptin modulates vascular tone and trophoblast function^42^.

In the context of CVD, the RAAS plays a significant role not only in disease progression but also in normal cardiovascular and kidney development. Notably, the angiotensin-converting enzyme/angiotensin-II/angiotensin-II type-1 (AT-1) receptor axis and the counter-regulatory angiotensin-converting enzyme 2/angiotensin-(1-7)/Mas receptor pathway are central to the development and function of the cardiovascular and renal systems^43^. A shared maternal-foetal molecular pathway involving AT-1 agonistic autoantibodies may underlie the elevated lifetime cardiovascular risk observed in PE women and their offspring. AT-1 agonistic autoantibody activity, measured by neonatal cardiomyocyte bioassay, was significantly increased in both maternal and foetal samples from preeclamptic pregnancies compared to controls (mother.17.5 ± 2.2 vs. 0.05 ± 0.4 Δbpm; foetus: 14.5 ± 1.8 vs. 0.5 ± 0.5 Δbpm)^44^. These findings support the role of in utero AT-1 agonistic autoantibody exposure in programming future cardiovascular function. Several studies indicate that AT-1 agonistic autoantibodies contribute significantly to the pathophysiology of PE by triggering vasoconstriction, elevating blood pressure, and enhancing coagulation processes^45^. Consequently, assessing the imbalance of regulatory factors involved in peripheral cardiovascular adaptation during pregnancy is key.

### Cardiovascular programming in the offspring of PE pregnancies

While the vascular and cardiac adaptations in the mother during PE have been widely studied, it is critical to address the impact the disease may have on foetal cardiovascular development. Central to this maternal-foetal interplay is the placenta^46^. It not only reflects maternal pathology but also actively influences foetal growth and cardiovascular programming through disrupted nutrient delivery, oxygenation and hormonal signalling ^23,35,47^. The foetal origins hypothesis proposes that adaptations made by the foetus in response to undernutrition can have a profound impact on the susceptibility to future disease in adulthood^48^. Central to this process is a ‘maternal-placental-foetal axis’, which integrates maternal adaptations and placental regulation to guide foetal development, ultimately shaping long-term health outcomes in the offspring as described by the Developmental Origins of Health and Disease Framework^23,48^. Increasing evidence suggests that exposure to PE during early life increases the risk of noncommunicable diseases, including CVD, in adulthood^23^. 1^17^. These findings appear to be largely independent of preterm birth or small-for-gestational-age (SGA) status^17^. Nonetheless, premature birth is a common effect of PE and may independently worsen outcomes in offspring; whether their combined effect further elevates long-term cardiovascular risk remains unclear^49^. This highlights the need to investigate potential structural alterations in the hearts of affected children to add a critical dimension to our understanding of these risks. Neonates born to mothers with PE exhibited reduced intraventricular septum thickness alongside significantly enlarged lumen diameters of the left, right and main coronary arteries, as well as the mitral, tricuspid, aortic and pulmonic valves within 48 h of birth. This distinct coronary dilation may serve as a marker of neonatal endothelial inflammation severity resulting from maternal PE^50^. Children of PE pregnancies showed increased late diastolic velocity (A’ wave) at mitral valve attachments, which was accompanied by decreased heart size, at 5–8 years old^51^. Despite reported associations in several studies, the findings lack consistency, and the underlying mechanisms remain unresolved. A recent study by Dimopoulou et al., which was published in 2024, found that while early childhood assessments of offspring exposed to hypertensive disorders of pregnancy, either gestational hypertension or preeclampsia, have reported increased relative wall thickness and elevated left ventricular mass compared with controls, these differences did not persist after adjustment for confounders^52^.

Collectively, these findings highlight the complexity and variability of cardiovascular outcomes following PE exposure. Given these long-term health implications, it is crucial to implement coordinated follow-up care for preeclamptic mothers and their children within both paediatric and obstetric health monitoring systems. Such integrated follow-up can facilitate early detection, prevention and management of cardiovascular and metabolic disorders, enabling timely interventions that may mitigate adverse outcomes across the lifespan. Understanding the underlying molecular mechanisms within the maternal-placental-foetal axis is essential to elucidate how this interplay predisposes both to long-term CVD risk.

## Genomics

### Genetic links between PE and CVD

PE is associated with both pre-existing and future CVD risk in affected women^19^. However, the complication is not only limited to the mother; offspring of preeclamptic women also face a heightened risk of developing CVD later in life^53^. Alsnes et al. suggest that shared genetic or lifestyle factors may contribute significantly to this association. Offspring exposed to PE during pregnancy exhibit more adverse cardiovascular risk profiles in young adulthood compared to those from normotensive pregnancies^54^. Familial predisposition has been well documented, with evidence pointing to a shared role of the maternal and foetal genome^55^. Overall, PE heritability is estimated to be 55%^56,57^.

Genome-wide studies have tried to decipher the underlying shared mechanisms between CVD and PE in affected mothers, with a particular focus on examining their shared genetic architecture^56^. They are based on the premise that many common diseases have a complex polygenic foundation, where numerous variants with modest individual effects collectively contribute to disease risk. Polygenic risk score analysis demonstrated that blood pressure polygenic risk scores are associated with increased risk of PE, as well as recurrent PE, severe forms of PE and higher blood pressure levels during pregnancy. Furthermore, blood pressure polygenic risk scores have also been associated with the more severe forms of PE^58^. Interestingly, Jouko et al. suggest that blood pressure polygenic risk scores could capture the genetic architecture better than the current preeclampsia-specific polygenic risk scores^59^. Notably, the genetic risk appears bidirectional; variants predisposing to hypertensive pregnancy disorders are also associated with increased atherosclerotic CVD risk^60^. Steinthorsdottir et al. conducted an analysis that identified five maternal genetic variants (*ZNF831/20q13, FTO/16q12, MECOM/3q26, FGF5/4q21* and *SH2B3/12q24*) associated with PE, all linked to blood pressure regulation. These genes are implicated in a range of shared risk factors such as obesity, hypertension, inflammation-related vascular dysfunction and vascular remodelling^61–63^. This highlights the importance of incorporating relevant genetic factors of metabolic dysfunction when analysing the intersectionality between CVD and PE. In addition to vascular risk, emerging evidence suggests a potential genetic predisposition to cardiomyopathy in women with PE. Gammill et al. reported that women who develop PE are more likely to carry protein-altering mutations in genes associated with cardiomyopathy, particularly *TTN* ^64^.

### Foetal genetic drivers of PE and the sFlt-1-cardiovascular axis

While most research in the past decade has focused on the maternal genome, the foetal contribution to PE remains underexplored. The only significant foetal variant found by Steinthorsdottir et al. was near the *FLT1* (*rs4769613*; *P* = 5.4 × 10^−11^) gene, which encodes Fms-like tyrosine kinase 1. This variant is biologically relevant, as its placental isoform, sFlt-1, plays a key role in PE pathology. Foetal variants may contribute to PE susceptibility, in line with inheritance patterns that involve both maternal and paternal genetic factors^65^. Analysis of both the foetal and maternal genomes identified independent *loci*, with the foetal *FLT1* variant showing no maternal effect, thus highlighting the critical role of foetal-specific genetic risk factors in PE development^55,57^. Another study further explored the association of this lead genetic *locus* having different effects on PE subtypes and found that the *rs4769613* allele occurred more frequently in pregnancies with LO-PE and non-SGA infants^57^. Additionally, prolonged exposure of the maternal vasculature to elevated levels of sFlt-1 and other antiangiogenic factors may contribute to lasting vascular alterations, potentially increasing the risk of CVD later in life^27^. A mouse model combining a high-fat diet before pregnancy with sFlt-1-induced PE revealed sex-specific changes in the vascular function of the adult offspring. These changes were observed in the contractile properties of the carotid artery^66^. Consequently, these findings underscore how combined maternal risk factors, including metabolic disease and elevated sFlt-1 exposure, could contribute to adverse cardiovascular programming in the offspring. Furthermore, elevated levels of sFlt-1 have been associated with atherosclerosis and HF, where circulating sFlt-1 serves as a marker of myocardial injury^67,68^. Nevertheless, the role of sFlt-1 in the subsequent development of CVD remains unclear, and further omics studies are required to elucidate its contribution in the context of PE.

## Epigenomics

### Epigenomic insights into PE and cardiovascular risk

Epigenetics has emerged as a critical area of study in understanding the interplay of environmental influences and genetic factors that shape disease development. In this review, epigenetics will be defined as ‘the study of molecules and mechanisms that can perpetuate alternative gene activity states in the context of the same deoxyribonucleic acid (DNA) sequence’^69^. Epigenetic mechanisms such as DNA methylation, histone modification and non-coding ribonucleic acids (RNA) are central to the pathophysiology of both PE and CVD. Epigenetic changes, involving key regulatory pathways, can often occur early in PE disease development and may serve as valuable biomarkers^70,71^. They are heritable DNA modifications that can influence gene expression without changing the DNA sequence. Interestingly, they are highly responsive to environmental and intrauterine factors such as those encountered during preeclamptic pregnancies^72^. The foundational work of Dr David Barker pioneered the concept of foetal programming, suggesting that adverse intrauterine conditions can predispose individuals to chronic disease, more specifically CVD, in adulthood^23^. Epidemiological studies have since supported this hypothesis, illustrating links between birth weight, a proxy for foetal environment, and long-term cardiometabolic health^73^. Parts of this developmental programming may be mediated through epigenetic modifications. In recent studies, the role of epigenetic modifications in foetal growth and long-term disease risk has been further explored. A placental epigenome-wide association study of birthweight in a diverse cohort of pregnant women revealed DNA methylation at 15 Cytosine-phosphate-Guanine (CpG) sites significantly associated with birthweight. Four of these CpG sites are linked to the expression of genes involved in lipid metabolism, inflammation, and oxidative stress, key factors in the development of CVD. Methylation at around one-third of the CpG sites is associated with birthweight and influences the expression of adjacent genes in the placenta^74^. More specifically, another study found that hypermethylation of CpG sites within *AluY* elements in trophoblasts was associated with reduced *ZNF554* expression. Subsequently, this might impair trophoblast invasion and contribute to the development of FGR^14^. These results indicate that DNA methylation in the placenta at *loci* involved in regulating birthweight may be linked to maternal perinatal cardiometabolic health and the increased risk of chronic diseases in offspring later in life.

### cfDNA analysis and epigenetic approaches in early screening for PE and maternal cardiovascular risk

Precise and early screening methods are essential for mitigating risk in PE. Liquid biopsy analysis of placenta-derived cell-free DNA (cfDNA) in the maternal plasma and associated methylation patterns has gained considerable attention^71^. cfDNA, short fragments of double-stranded DNA released from cells undergoing cell death or dividing and found in many fluids, including blood, offers a non-invasive insight into the epigenetic landscape of PE^71,75^. The continuous release of placenta-derived materials, including cell-free RNA and cfDNA, begins early in pregnancy and, as a result, could serve as an early marker for disease detection^76^. Early PE screening is crucial for mitigating long-term cardiovascular health risk in the high-risk population. Low-dose aspirin prophylaxis (150 mg) in pregnancy has been shown to provide a significantly lower incidence of PE in pregnant women in the ASPRE trial^77^. While low-dose aspirin has demonstrated cardioprotective effects and is well-established in secondary prevention, its role in primary prevention during pregnancy remains controversial, and knowledge available about its long-term impact on cardiovascular health is limited^78^. At present, the most common screening tool for PE in the first trimester is the Fetal Medicine Foundation Algorithm, which can predict 90% of EO-PE cases and 75% of preterm-PE cases^79^. However, the first trimester PE screening involves multiple factors, making it complex and not universally applicable. Adil et al. propose that cfDNA nucleosome profiling could reveal early placental ($\le 16$ weeks) and endothelial tissue abnormalities, offering a simpler approach to predicting PE risk with just the two clinical parameters, blood pressure and BMI. Individuals who develop PE have significantly lower placental tissue contribution and higher endothelial tissue contribution compared to those with normal pregnancies, revealing that early tissue dysfunction may reflect placental and maternal endothelial dysfunction^76^. Furthermore, De Borre et al.^71^ also explored cfDNA methylation potential and refined a differential cfDNA methylation analysis for EO-PE prediction. It was validated through whole-genome bisulfite sequencing, revealing PE-associated methylation patterns that are consistent with target-enrichment bisulfite sequencing. Integrating this cfDNA methylation approach with routinely available maternal risk factors culminates in a combined risk score that accurately predicts 72% of EO-PE cases at 80% specificity^71^. While existing prediction models have identified potential biomarkers for PE, their associations with adverse outcomes necessitate further research to fully comprehend the impact of epigenetic modifications on cardiovascular health and differences in maternal-foetal tissue composition, especially concerning gender-specific outcomes in offspring^76^.

## Transcriptomics

### Transcriptome profiling for molecular subtyping of PE

Over the past decade, transcriptional subtypes of PE have been conceptualised to gain deeper insight into underlying disease mechanisms. Central to these efforts is transcriptomics, the large-scale study of the transcriptome, the complete set of all expressed RNA species^80^.Technological advances in genome sequencing have facilitated the exploration of the transcriptome through tools such as microarrays and next-generation sequencing, both in the placental tissue and in the maternal peripheral blood^80–82^. Leavey et al.^80^ pioneered the exploration of subtyping the placental transcriptome of preeclamptic women and identified three distinct molecular subclasses that differ in clinical and molecular features. (1) ‘canonical/placental’ subtype involves preterm onset, abnormal uterine Doppler ultrasound waveforms indicative of uteroplacental insufficiency^83^, low birthweight, high sFlt-1 levels, and high endoglin expression; (2) ‘maternal’ subtype presents at term with appropriate birthweight and no placental vascular lesions; and (3) ‘immunological’ subtype is marked by SGA neonates, low placental weight, and immune dysregulation^80^. Interestingly, two placental subtypes of normotensive SGA births present transcriptional and histopathological similarities to the ‘canonical’ and ‘immunological’ PE subtypes, suggesting shared pathophysiological mechanisms^84^. This is clinically relevant, as studies have demonstrated that women with PE and coexisting foetal growth disturbance exhibit the most severe cardiovascular dysfunction, regardless of gestational age at onset^32,33^. Despite these advancements, the relationship between placental transcriptional subtypes of PE and the long-term CVD risk remains largely underexplored. Consequently, it is important to recognise the heterogeneity of the disease to further explore the role of maternal cardiovascular health in PE.

### Single-cell RNA and cell-free RNA profiling in PE: insights into pathogenesis

Single-cell RNA- and single-nucleus RNA-sequencing are powerful tools to analyse gene expression at a very high resolution, either at the level of single cells or isolated nuclei^85,86^. A recent single-cell RNA- and single-nucleus RNA sequencing study of placental tissue by Admati et al. demonstrated that EO-PE and LO-PE follow a distinct pathophysiology. EO-PE displays dysregulation of sFlT-1/PlGF, cellular stress and pre-apoptotic states in the vasculature, while LO-PE exhibits minimal involvement in inflammation or angiogenesis. Together, these findings denote that EO-PE and LO-PE forms exhibit distinct pathophysiological mechanisms, cementing EO-PE as the more placentally manifested disease^81^. A plasma-based cell-free RNA analysis (18–22 weeks) by Elovitz et al.^87^ has refined this stratification and identified two refined molecular subtypes of hypertensive disorders of pregnancy: placental-associated mapping clinically to severe/preterm PE and immune-associated mapping clinically to mostly term PE and gestational hypertension. The study revealed that the placental gene *PAPPA2* strongly predicts severe PE in individuals without pre-existing high-risk factors, including prior cardiovascular risk, months before symptoms appear, with overexpression correlating with earlier delivery. Unexpectedly, the placental-associated, hypertensive disorders of pregnancy are most predictive in individuals without high-risk factors, for whom effective risk assessment tools remain largely unavailable^87^. Additionally, a study utilising integrative systems-biology approaches found through weighted gene co-expression network analysis that *PAPPA2* and *FLT1* belong to the same dysregulated co-expression module in PE and are strongly co-expressed. Genes within this module were significantly overrepresented among those that correlated with mean arterial pressure^14^. This is further supported by Hamdan et al., who identified *FLT1, HTRA4, LEP* and *PAPPA2* as hub genes for PE through bioinformatic analysis^88^. These findings suggest that both *PAPPA2* and *FLT1* may contribute to the anti-angiogenic state and hypertension observed in severe PE. Ultimately, meta-analytic evidence suggests that EO-PE, as well as LO-PE are associated with risk factors for adverse maternal cardiovascular events later in life, with EO-PE conferring a greater long-term burden^89^.

### Placental angiogenesis and foetal cardiovascular programming: a transcriptomic perspective

Levels of sFlt-1/PlGF have been linked to cardiovascular outcomes in PE patients. Shahul et al. found that changes in global longitudinal strain correlate with circulating levels of PE biomarkers, including sFlt-1 and soluble endoglin. Circulating sFlt-1 levels have also been associated with left ventricular mass index, a marker of cardiac remodelling^90^. These findings underscore the necessity of further transcriptomic analysis of both the placenta and the maternal peripheral blood to fully elucidate the dynamic interplay between the placenta and the maternal cardiovascular system. Additionally, transcriptomic analysis not only provides valuable insights into maternal cardiovascular health but also provides a framework for exploring implications for cardiac foetal programming in PE pregnancies. In PE, reduced levels of the pro-angiogenic PlGF and elevated levels of the anti-angiogenic factor sFlt-1 in the maternal peripheral blood are associated with impaired placental function, FGR, preterm birth and adverse neonatal outcomes^91,92^. Crucially, PlGF has been identified as a key player in embryonic heart development, and decreased maternal PlGF levels at 11–13 weeks’ gestation have been linked to cases of isolated foetal heart defects^93^. A recent single-cell RNA sequencing analysis has explored PlGF’s role in cardiovascular development and regeneration with data derived from human and primate embryonic hearts. PlGF exhibited a biphasic expression pattern, first in heart progenitors and later in vascular cells. Furthermore, the application of *PlGF* modRNA during cardiomyocyte differentiation enhanced the production of both cardiomyocytes and endothelial cells, while *PlGF* deletion reduced their generation in vitro^94^. Thus, this suggests that PlGF is vital for cardiac and vessel development, and its disruption in PE may increase the risk of adverse cardiovascular outcomes in children, warranting further transcriptomic analysis^17^.

## Proteomics

### Proteomic profiling for identifying molecular subtypes of PE

Integrating multi-omics approaches has significantly advanced the understanding of PE heterogeneity. Proteomics is the large-scale study of protein expression, structure, modification and interactions^95^. It provides functional insights that complement genomic and transcriptomic data by linking molecular changes to the phenotype^95^. Building on the transcriptomic subtypes identified by Leavey et al.^80^, Than et al. employed proteomic data to propose a fourth molecular subclass, the ‘metabolic’ subtype^96^. Proteomic analysis of maternal plasma from PE cases and controls revealed four distinct subclasses: (1) a ‘metabolic’ subtype predominantly associated with EO-PE and SGA neonates, characterised by metabolic and prothrombic alterations, low maternal BMI, and high maternal and foetal malperfusion; (2) a ‘maternal anti-foetal immunity’ subtype, marked by recurrent PE, predominantly LO-PE, chronic placental inflammation, proinflammatory proteomic signatures and low incidence of SGA and high BMI; (3) an ‘extracellular matrix (ECM)-related’ subtype, linked to LO-PE and high birthweight, displaying minimal proteomic changes and ECM dysregulation and mildest phenotype; and (4) a ‘placental’ subtype, defined by severe EO-PE, with high maternal BMI and rates of chronic hypertension, the strongest anti-angiogenic imbalance, largest proteomic changes, and the lowest neonatal birthweights^80,96,97^. Proteomic subtypes could capture the diverse biological mechanisms underlying PE and may offer critical insights into the distinct cardiovascular trajectories associated with a molecular profile. Certain PE subclasses are associated with clinical risk factors, including high maternal BMI and chronic hypertension, which may increase susceptibility to future cardiovascular conditions^3^. Nonetheless, beyond these well-established clinical markers of disease susceptibility, proteomics analyses reveal complex maternal and placental contributions^97^. Platelet activation is a common dysregulated pathway across all subclasses, suggesting a central role of platelet-driven inflammation and thrombosis in PE. Platelets are heterogeneous, and specific subpopulations have been implicated in CVD, particularly acute coronary syndromes, due to their contribution to thrombotic risk^98,99^. This suggests a potential shared mechanism between preeclampsia and CVD, wherein platelet hyperreactivity may contribute to both placental and vascular injury^100^. Notably, the two most severe subclasses (1) metabolic and (4) placental, both involve maternal vascular malperfusion, albeit through divergent mechanisms. In the metabolic subtype, disordered coagulation leads to maternal and foetal vascular lesions and placental abruption. In contrast, in the placental subtype, the trophoblast dysfunction is affecting primarily the maternal compartment. Consequently, beyond general risk assessment, proteomics frameworks may help uncover CVD pathways in women after PE by exploring both the placental and maternal contributions to long-term CVD risk. Studying coagulation and prothrombotic mechanisms within molecular PE subgroups could enhance cardiovascular risk prediction and uncover tailored pharmacotherapeutic strategies^97^. Proteomic subtyping highlights the complexity and heterogeneity of PE and offers a valuable framework by linking PE molecular profiles to cardiovascular outcomes. However, as the molecular subtyping of PE is a relatively recent advance and CVD has a long-term progression, there is currently limited clinical data on cardiovascular health available to definitively validate these associations. Thus, further longitudinal studies are required to confirm their prognostic significance for long-term cardiovascular risk evaluation.

### Proteomic distinctions and cardiovascular pathways in PE

Current data on the proteome in women following PE remains limited, particularly from studies with significant sample sizes to enable robust subtype classification. To demonstrate proteomic distinctions and the contribution of cardiovascular pathways, Lindley et al. analysed plasma samples collected 48 h before delivery from patients diagnosed with EO-PE and LO-PE. Their findings demonstrated that variations in circulating proteins are linked to pathways involved in CVD, including angiogenesis, blood pressure regulation, cell adhesion, inflammation and metabolism^101^. These processes may reflect key mechanisms in CVD and appear to be implicated in emerging transcriptomic and proteomic analyses of molecular PE subtypes^80,96,97^. Although generally consistent patterns across the cardiometabolic proteome are observed in both EO-PE and LO-PE, several proteins associated with cardiovascular stress and inflammation are more pronounced in patients with EO-PE diagnosed before 34 weeks of gestation^101^. The greater expression of these proteins in EO-PE cases aligns with clinical evidence indicating more severe maternal vascular compromise and a heightened risk of future CVD in this group of patients^29,102^. Nevertheless, comprehensive multi-omics studies across disease states are required to clarify whether the placental transcriptional activity contributes to the circulating cardiovascular proteome and the pathogenesis of CVD^101^.

### Proteomic signatures of vascular dysfunction and senescence in peripartum cardiomyopathy and PE

Cardiovascular maladaptation in pregnancy can manifest in various forms, one of the most severe being peripartum cardiomyopathy. It is a life-threatening subtype of HF towards the end of pregnancy or in the months following delivery^103^. Increasing evidence implicates vascular dysfunction as a central mechanism in peripartum cardiomyopathy, characterised by left ventricular systolic impairment and clinical HF^104^. Shared genetic predispositions, particularly mutations in *TTN*, are linked with increased susceptibility to both PPCM and PE, suggesting overlapping molecular underpinnings between these conditions^64^. In a proteomic study, Roh et al. identified the senescence-associated secretory phenotype as the most highly upregulated pathway in women with peripartum cardiomyopathy or PE. It consists of a collection of proteins primarily secreted by senescent cells and serving as a marker of biological ageing. Placentas from preeclamptic women exhibit multiple markers of amplified senescence and tissue ageing. This is accompanied by elevated gene expression of 28 circulating proteins in serum samples of PE and peripartum cardiomyopathy patients, contributing to enrichment of the senescence-associated secretory phenotype pathway^105^. Targeted biomarker analysis by Suvakov et al. uncovered that women with a history of PE exhibit a fourfold increased risk of major cardiovascular events over an average 6-year follow-up period, alongside a more unfavourable profile of senescence and cardiovascular biomarkers. This includes reduced urinary α-Klotho, elevated leptin levels, a higher leptin/adiponectin ratio, and increased circulating extracellular vesicles positive for tissue factor^106^. Other studies have elucidated the role of leptin in PE, exploring its importance in serving as a link between placental dysfunction and metabolic disease. Elevated periconceptional leptin levels were associated with hypertensive disorders and gestational diabetes, even after controlling for age and BMI^107^. Microarray analysis has identified *LEP* as one of the most overexpressed genes in the placenta of women with EO-PE^108^. While evidence on the impact of maternal leptin levels on the offspring remains inconclusive, elevated *LEP* expression has been observed in SGA placentas relative to controls, suggesting a potential role in altered foetal growth trajectories^109^. These findings highlight the critical interplay between metabolic disruptions and placental disease, which may serve as a key link to CVD. Overall, mounting evidence suggests that diverse cellular stressors, including DNA damage, oxidative stress, ischaemia and other environmental insults across these conditions, can accelerate placental senescence, particularly within the context of PE^110^.

## Metabolomics

Metabolomics enables the systematic identification and quantification of all metabolites in a given organism or biological sample and has emerged as a powerful tool for assessing metabolic signatures^111^. Metabolic disorders, including obesity and insulin resistance, are well-established risk factors for PE. Obesity (BMI > 30 kg/m^2^), is a significant risk factor for both EO-PE and LO-PE, with a notably increased risk of LO-PE forms in overweight (BMI > 25 kg/m^2^) and obese women^112^. Variables like chronic hypertension and advanced maternal age can further elevate risk^113^. Beyond pregnancy, women with a preeclamptic history exhibit lasting metabolic disturbances, higher blood pressure levels, increased BMI and greater insulin resistance compared to unaffected women^114^. Obesity directly promotes the development of cardiovascular risk factors such as dyslipidaemia, type 2 diabetes, hypertension and sleep disorders and is also an independent contributor to the onset and mortality of CVD^115^. These metabolic disturbances drive systemic inflammation, endothelial dysfunction and placental malperfusion^116^. In LO-PE, metabolic alterations identified before 24 weeks of gestation predominantly reflect maternal overweight or obesity. By 30-32 weeks, these alterations become increasingly characteristic of PE itself. At term, women with PE exhibit marked increases in circulating total lipids, very-low-density lipoproteins, triglycerides and total fatty acids^117^.

### Metabolomic insights into PE: linking lipid dysregulation, oxidative stress and cardiovascular risk

Metabolomic profiling examines the metabolome, the complete set of small molecules produced by cells, and provides critical insights into the underlying metabolic dysregulation linking PE and CVD^118^. Recent metabolomics studies have uncovered profound alterations in the lipid profiles of patients with severe PE, shedding light on potential mechanistic links between oxidative stress and disrupted lipid metabolism^74,119,120^. While metabolomics has been investigated as a predictive tool for PE in several studies^118,121–123^, the relationship between the maternal metabolome and adverse pregnancy outcomes remains incompletely defined. Idler et al. were the first to investigate this, identifying significant metabolic disruptions in sphingolipid metabolism, methylhistidine metabolism, and phosphatidylethanolamine biosynthesis in PE cases with adverse outcomes via direct injection liquid chromatography-mass spectrometry combined with nuclear magnetic resonance spectroscopy^119^. Additionally, a study by He et al. found a strong association between oxidised phospholipids in the serum metabolome of preelcmaptic women, likely driven by hypoxia-induced oxidative stress. Furthermore, consistent decreases in lipid species, including lysophosphatidylcholine, phosphatidylcholine and lysophosphatidylethanolamine in the serum of preeclamptic women, point to a phospholipid-centred metabolic axis, with implications for insulin signalling, immune responses, and membrane homoeostasis^120^. This may contribute to the elevated cardiovascular risk observed in women with PE, as prolonged or excessive exposure to oxidised phospholipids can drive chronic inflammation and promote atherogenesis^124^. Women with a history of PE have a significantly increased risk of early-onset atherosclerosis compared to those with normotensive pregnancies^125^. Phospholipids are primary targets of oxidation in low-density lipoprotein particles^126^. In an atheroprone low-density lipoprotein receptor-deficient mouse model, sFlt-1 induced PE-amplified atherosclerotic plaque inflammation without affecting plaque size or traditional risk factors. This heightened inflammation, despite similar plaque size, may explain the two-fold increased risk of heart attack and stroke in women after PE, since plaque inflammation links to rupture and ischaemic events in humans^127^. Statins, inhibitors of 3-Hydroxy-3-Methylglutaryl-Coenzyme A reductase, have been explored as potential pharmacological interventions for PE due to their ability to reduce lipid levels and modulate endothelial injury and inflammation^128^. There is conflicting evidence on the benefit of statin use in PE, and Döbert et al. elucidated that Paravastatin treatment in high-risk women (35–37 weeks of gestation until delivery or 41 weeks) does not reduce PE incidence^129^. Statins are well-established in CVD prevention, with the United States Preventive Services Taskforce suggesting a potential benefit for adults aged 40–75 with CVD risk factors^130^. Additionally, they prevent chemerin upregulation in PE, potentially via nitric oxide and low-density lipoprotein receptor pathways, and may improve the sFLT-1/PlGF ratio^131^. Combining omics approaches could help identify individuals most likely to benefit from potential therapeutic interventions, such as statin therapy, paving the way for personalised interventions in PE management.

### Fatty acid oxidation in PE and HF

Oxidative stress is a known contributor to impaired fatty acid oxidation; a process increasingly acknowledged in the pathogenesis of PE. Recent metabolomic research demonstrated that women diagnosed with PE exhibit elevated levels of several altered metabolites in their circulation, with the most pronounced changes in L-cystine, L-cysteine, L-acetylcarnitine, carnitine and D-alpha-aminobutyric-acid^122^. Fatty acid ß-oxidation is the primary metabolic pathway for the oxidation of fatty acids, serving as the predominant energy source for both the heart and skeletal muscle^132^. In HF linked to diabetes and obesity, myocardial fatty acid oxidation is elevated, whereas in HF associated with hypertension and ischaemia, it is reduced^133^. The precise role of fatty acid oxidation in PE remains unclear, and forms of HF in PE, including those linked to metabolic and vascular dysfunction, remain underexplored^134^.

### Glycosylation alterations and galectins in PE

Altered glycosylation patterns, including changes in N-linked glycosylation, the attachment of oligosaccharides to asparagine or arginine, and O-linked glycosylation, which occurs mainly at serine and threonine, have been observed in women with PE^135–137^. Placental endoplasmic reticulum stress, as observed in EO-PE, drives the secretion of mis-glycosylated proteins. Consequently, normal biological functions are impaired, contributing to maternal metabolic dysregulation^136^. DNA sequencer-assisted fluorophore-assisted carbohydrate electrophoresis analysis of placental tissue from preeclamptic women revealed significant changes in N-glycosylation of placental membranes, including a reduction in fucosylation and an increase in paucimannosidic structures, and decreased sialylation of the insulin receptor^138^.

Protein glycosylation, particularly of the terminal sialic acid linkages, is pivotal in regulating both pro-inflammatory and anti-inflammatory immune responses^139^. These alterations in glycosylation can contribute to vascular dysfunction and inflammation, a hallmark of PE^140^. Galectins, a family of glycan-binding proteins, have been studied in the context of PE, CVD and metabolic disorders due to their significant roles in immune modulation and cellular signalling. Dysregulation of Galectin-1 has been linked to PE^141,142^, while galectin-3 has been affiliated with HF and cardiac fibrosis^143,144^. Xie et al. demonstrated in a galectin-1-deficient mouse model that changes in placental glycosylation, particularly through Sda-capped N-glycans, impair trophoblast invasion^145^. This disruption in trophoblast invasion interferes with spiral artery remodelling, a crucial process in the pathogenesis of PE^146^. Furthermore, galectins have also been investigated in metabolic regulation. A metabolomic approach was employed in the POEM study to examine the role of galectin-1 and galectin-3 in lipid metabolism. Galectin-1 is associated with adiposity, insulin secretion, and insulin sensitivity, while galectin-3 correlates with variances in the triglyceride-glucose index and fasting insulin levels^147^. Nevertheless, evidence for specific alterations in glycosylation patterns in PE remains limited, particularly in human biological samples. Additional investigation of glycosylation patterns is essential to clarify the role of the glycan-galectin-heart axis in the pathophysiology of PE.

### Microbiomics approaches in PE and CVD

Gut dysbiosis has been implicated in the pathogenesis of both PE and CVD, and microbiomics, the detailed study of molecules responsible for the structure, function and dynamics of a microbial community, has revolutionised our understanding of these host-microbe ineractions^148–150^. Short-chain fatty acids (SCFAs), such as butyrate, propionate, and acetate, are microbial byproducts. Their levels in the gut and other tissues can be influenced by environmental factors, including diet and antibiotics^151^. Jin et al. utilising a targeted metabolomics and 16S rRNA sequencing approach, revealed a significant reduction of SCFA-producing bacteria and SCFA in the stool of PE women. Substantially, the gut microbiota of PE patients exacerbated the pathology and symptoms in a preeclamptic rat model, whilst the microbiota of healthy women had significant protective effects^148^. *A. muciniphila* and propionate/butyrate significantly alleviated pathologies in this animal model by promoting autophagy and M2 macrophage polarisation in the placenta^148^. Propionate significantly attenuated cardiac hypertrophy, fibrosis, vascular dysfunction, and hypertension in two mouse models of hypertensive cardiovascular damage, highlighting its potential protective mechanisms^152^. Investigating the role of SCFAs in blood pressure regulation, the HELIUS study revealed that while faecal microbiota composition is associated with blood pressure, there are strongly divergent associations between ethnic groups^153^. Microbiota have been linked to health disparities, with specific taxa exhibiting recurrent associations with ethnicity^154^. This raises the possibility that similar ethnic differences could shape susceptibility to HPD, including PE. Emerging evidence points to ethnic disparities in PE incidence, particularly among racial and ethnic minority groups in the US^155^. However, it remains unclear to what extent these disparities are driven by socioeconomic factors, biological differences, or their intersection^156^. Crucially, Countouris et al. observed racial and ethnic differences in the cardiology follow-up care after PE in the United States, likely influenced by social determinants of health^157^. These disparities highlight broader structural inequities in healthcare access and underscore the need for targeted, system-level interventions to improve postpartum care for both women and children. Advancing personalised medicine frameworks to address racial disparities requires a deeper understanding of the complex interplay between biology and the environment. The integration of microbiomics with other systems-biology approaches offers a transformative pathway to unravel the complex, multilayered determinants driving these inequities, with the goal of advancing health equity across diverse populations.

## Outlook

More research is needed to fully understand the pathophysiology of PE, the dynamics of cardiovascular adaptations during pregnancy, and the molecular signatures underlying maladaptive responses. Such efforts are essential for advancing our understanding of maternal and offspring health risks and long-term outcomes.

**Fig. 3:**
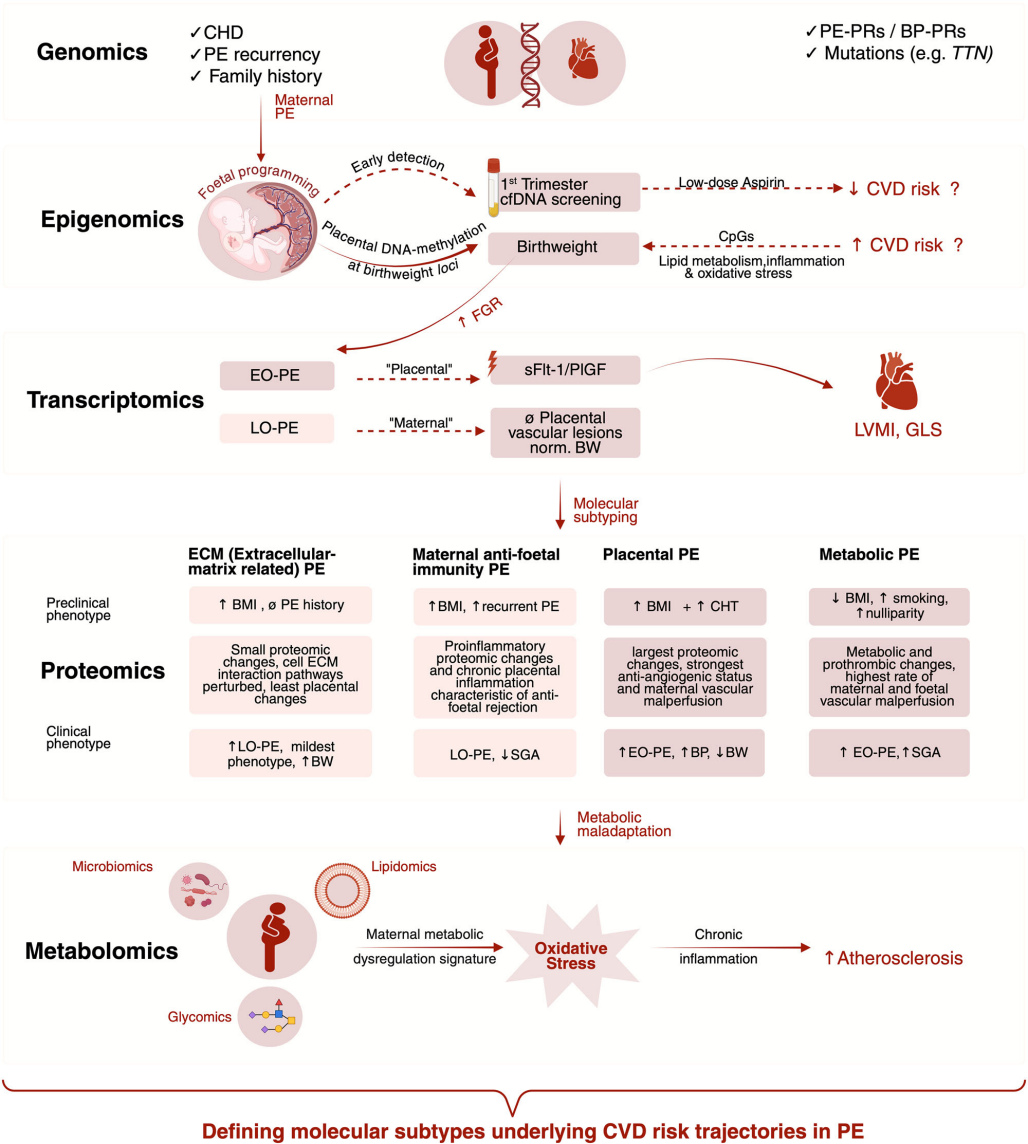
A molecular atlas of risk-linking PE to CVD. This figure proposes a molecular atlas linking preeclampsia (PE) to cardiovascular disease (CVD) risk through integrative multi-omics data, highlighting four proteomics-defined PE subtypes. They introduce a concept for more distinct characterisation beyond the classical early-onset (EO-PE) and late-onset (LO-PE) definitions. These subtypes—metabolic, maternal anti-foetal immunity, extracellular matrix related (ECM) and placental—capture distinct pathophysiological mechanisms underlying PE and their overlap with CVD pathways. Integrative systems-biology approaches provide a framework for understanding PE heterogeneity and identifying opportunities for integrative therapeutic interventions and personalised medicine approaches. BMI Body Mass Index, BP-PRs Blood Pressure Polygenic Risk Scores, BW Birth Weight, CHD Congenital Heart Defects, CHT Chronic Hypertension, cfDNA cell-free DNA, CpGs Cytosine-phosphate Guanine sites, CVD Cardiovascular Disease, EO-PE Early-Onset Preeclampsia, FGR Foetal Growth Restriction, GLS Global Longitudinal Strain, LVMI Left Ventricular Mass Index, LO-PE Late-Onset Preeclampsia, PE Preeclampsia, PE-PRs Preeclampsia Polygenic Risk Scores, PlGF Placental Growth Factor, SGA Small for Gestational Age, sFlt-1 Soluble Fms-like Tyrosine Kinase-1, TTN Titin.

This review highlights the complex pathophysiology of PE, due to the different subtypes and onset timing, and its established association with long-term cardiovascular risk across the maternal-placental-foetal axis^19^. Multi-omics approaches from genomics, epigenomics, transcriptomics, proteomics, metabolomics, and microbiomics offer powerful tools to dissect the complex pathophysiology of PE. Each layer contributes distinct yet complimentary insights into genetic risk, molecular regulation, protein function, metabolic disruption and microbiome-driven influences (Fig. 3). While the integration of these omics techniques uncovers the heterogeneity of PE, the molecular mechanisms linking PE to CVD in the mother and the offspring remain incompletely understood. Although research has predominantly focused on maternal health, growing evidence points to the critical role of in utero exposure to the altered intrauterine environment of PE in shaping disease susceptibility in the offspring^158^. Pregnancy is increasingly recognised not only as a physiological stress test for the maternal system but also as a critical window of vulnerability during which early-life exposures can have lasting biological effects^159^. This review emphasises the need for longitudinal follow-up studies of women with a history of PE and their offspring. PE may represent a significant early determinant of cardiometabolic risk in the offspring, potentially mediated by persistent alterations in vascular function^160^, endocrine signalling^158^, or epigenetic regulation^53^. Additionally, ethnic background and socioeconomic status are key factors when examining the developmental origins of diseases linked to PE^154,155,157^. A more integrative systems-biology approach considering both the clinical and molecular features of EO-PE and LO-PE is essential to elucidate how these subtypes differentially affect foetal programming and postnatal health trajectories. Incorporating these factors into predictive models can potentially enable the refinement of individual risk profiles and support the development of targeted and equitable preventive strategies for improving offspring and maternal health outcomes.
